# Exoskeleton assistance symmetry matters: unilateral assistance reduces metabolic cost, but relatively less than bilateral assistance

**DOI:** 10.1186/s12984-018-0381-z

**Published:** 2018-08-09

**Authors:** Philippe Malcolm, Samuel Galle, Pieter Van den Berghe, Dirk De Clercq

**Affiliations:** 1 Department of Biomechanics and Center for Research in Human Movement Variability, University of Nebraska Omaha, Omaha, NE 68182 USA; 2 Department of Movement and Sports Sciences, Ghent University, B-9000 Ghent, Belgium

**Keywords:** Exoskeleton, Symmetry, Asymmetry, Unilateral, Bilateral, Metabolic, Magnitude, Work, Ankle, Walking

## Abstract

**Background:**

Many gait impairments are characterized by asymmetry and result in reduced mobility. Exoskeletons could be useful for restoring gait symmetry by assisting only one leg. However, we still have limited understanding of the effects of unilateral exoskeleton assistance. Our aim was to compare the effects of unilateral and bilateral assistance using a within-subject study design.

**Methods:**

Eleven participants walked in different exoskeleton conditions. In the *Unilateral* conditions, only one leg was assisted. In *Bilateral Matched Total Work*, half of the assistance from the *Unilateral* conditions was applied to both legs such that the bilateral sum was equal to that of the *Unilateral* conditions. In *Bilateral Matched Work Per Leg*, the same assistance as in the *Unilateral* conditions was provided to both legs such that the bilateral sum was the double of that of the *Unilateral* conditions. In the *Powered-Off* condition, no assistance was provided. We measured metabolic energy consumption, exoskeleton mechanics and kinematics.

**Results:**

On average, the *Unilateral*, *Bilateral Matched Total Work* and *Bilateral Matched Work Per Leg* conditions reduced the metabolic rate by 7, 11 and 15%, respectively, compared with the *Powered-Off* condition. A possible explanation for why the *Unilateral* conditions effectively reduced the metabolic rate could be that they caused only very little asymmetry in gait biomechanics, except at the ankle and in the horizontal center-of-mass velocity. We found the highest ratio of metabolic rate reduction versus positive work assistance with bilateral assistance and low work per leg (*Bilateral Matched Total Work*). Statistical analysis indicated that assistance symmetry and assistance per leg are more important than the bilateral summed assistance for reducing the metabolic rate of walking.

**Conclusions:**

These data bridge the gap between conclusions from studies with unilateral and bilateral exoskeletons and inform how unilateral assistance can be used to influence gait parameters, such as center-of-mass velocity.

**Electronic supplementary material:**

The online version of this article (10.1186/s12984-018-0381-z) contains supplementary material, which is available to authorized users.

## Background

In healthy people, walking is generally a symmetrical movement. Studies often assume full gait symmetry for simplifying data processing by analyzing only one side of the body [1]. While some studies report asymmetry even in healthy walking [2, 3], gait asymmetry is mainly a concern in various gait impairments. Unilateral amputees have slower forward speeds and longer stance durations during an intact leg stance compared with a prosthetic leg stance [4]. Hemiparesis patients often display asymmetry in step length [5–7] and gait mechanics [8, 9], and the elderly often show asymmetry in trunk acceleration [10]. Restricted mobility due to wearing an orthosis can also lead to gait asymmetry [11]. In many rehabilitation programs, assessing gait symmetry is an important aspect of evaluating rehabilitation effectiveness [12].

Many devices have been proposed to reduce gait asymmetry. There are indications that ankle-foot orthoses can improve gait symmetry in stroke patients [13]. Powered prostheses have been developed and have been shown to renormalize the gait of unilateral amputees [14]. Training methods such as split-belt treadmill walking [15, 16] or walking with ankle weights [17] have been developed to help stroke survivors. Another technology that is rapidly evolving is the use of robotic exoskeletons [18]. In hemiparetic stroke patients, the ankle of the paretic side is thought to be primarily responsible for compensatory movements [19], which is why recent attempts to assist the gait of stroke patients have focused on assisting that side [20, 21]. In contrast with prostheses, most applications for exoskeletons focus on objectives other than gait symmetry (e.g., metabolic rate reduction [22–29], gait retraining [30, 31] and performance improvement [32]). Compared with such methods as split-belt treadmills or ankle weights, exoskeletons are an interesting new approach for restoring symmetry because they allow assistance with a specific timing [23–26] and magnitude [24, 28, 33] at specific joints.

However, there is still an incomplete understanding of how the effects of unilateral assistance at the ankle propagate through the rest of the body. Ankle exoskeletons are known to also indirectly affect the rest of the body, e.g., by providing hip assistance [24, 34–36] or center-of-mass rebound assistance [24, 33]. There have been multiple studies with unilateral [20, 21, 33, 37–40] and bilateral ankle exoskeletons [22, 24–26, 28, 29, 34, 36], but no studies have reported a within-subject comparison of unilateral and bilateral assistance. Thus, there is a lack of knowledge of whether different results between experiments are due to differences in exoskeleton hardware or controls. Understanding the differences between studies with unilateral and bilateral exoskeletons could improve this overall understanding and benefit applications of unilateral and bilateral exoskeletons.

Therefore, our aim was to compare the effects of unilateral and bilateral ankle exoskeleton assistance. Other experimental paradigms, such as a unilateral plantarflexion restriction [11] and an imposed asymmetric step frequency [41], have shown that gait asymmetry leads to increases in both the joint work and the metabolic rate. Based on these prior findings, we expected that unilateral exoskeleton assistance will still reduce the metabolic rate. However, we also expected that unilateral assistance will lead to asymmetry, mostly at the ankle, and will in turn lead to relatively smaller reductions in metabolic cost than bilateral assistance.

## Methods

### Participants

Thirteen healthy male volunteers participated. We retained data from eleven participants (21.3 ± 0.1 y, 74 ± 3 kg, 182 ± 2 cm, mean ± standard error). Data from two participants were excluded due to exoskeleton malfunctions. All participants provided informed consent. The protocol was approved by the ethical committee of the Ghent University Hospital.

### Exoskeleton hardware

**Fig. 1:**
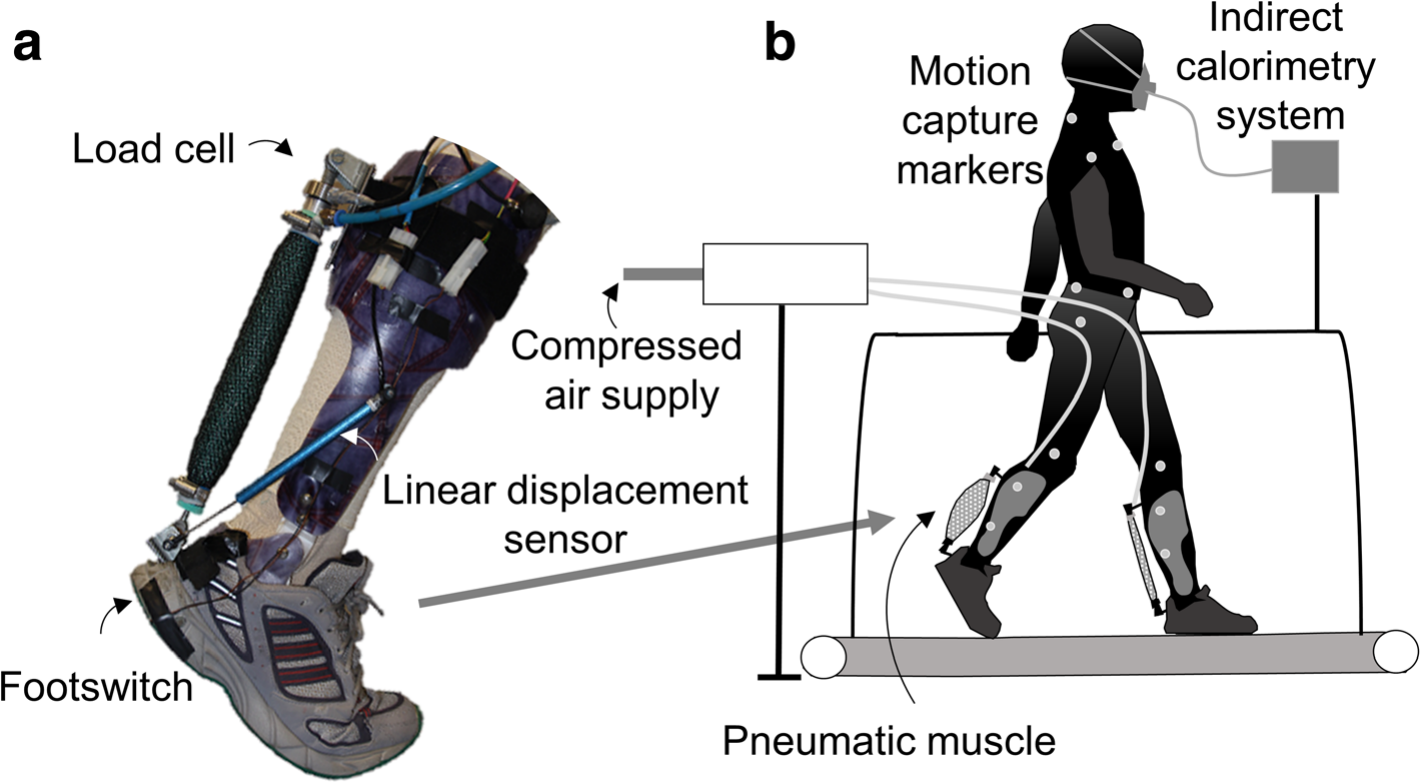
Experimental setup. **a** Exoskeleton. **b** Setup

We used bilateral exoskeletons similar to those we used previously ([26], Fig. 1). The exoskeletons consisted of a thermoplastic shell with a hinge joint at the ankle and pneumatic muscles that were 27 cm long and 3 cm in diameter. We adjusted the attachments of the pneumatic muscles such that they allowed 15° dorsiflexion at a low passive stretch force between 35 to 40 N. We mounted load cells (210 Series, Richmond Industries Ltd., Reading, UK) in series with the pneumatic muscles and a linear displacement sensor (SLS130, Penny&Giles, Christchurch, UK) between the foot and shank to monitor pneumatic muscle force, ankle angular velocity and moment arm, similar to [24]. The exoskeletons were worn with running shoes with foot switches mounted underneath the heels (Multimec 5E/5G, MEC, Ballerup, Denmark) to detect foot contact. The exoskeleton and sensors weighed 0.92 kg per side. The pneumatic muscles were connected via hoses to a valve station (CPE24, Festo, Esslingen am Neckar, Germany).

### Conditions

**Fig. 2:**
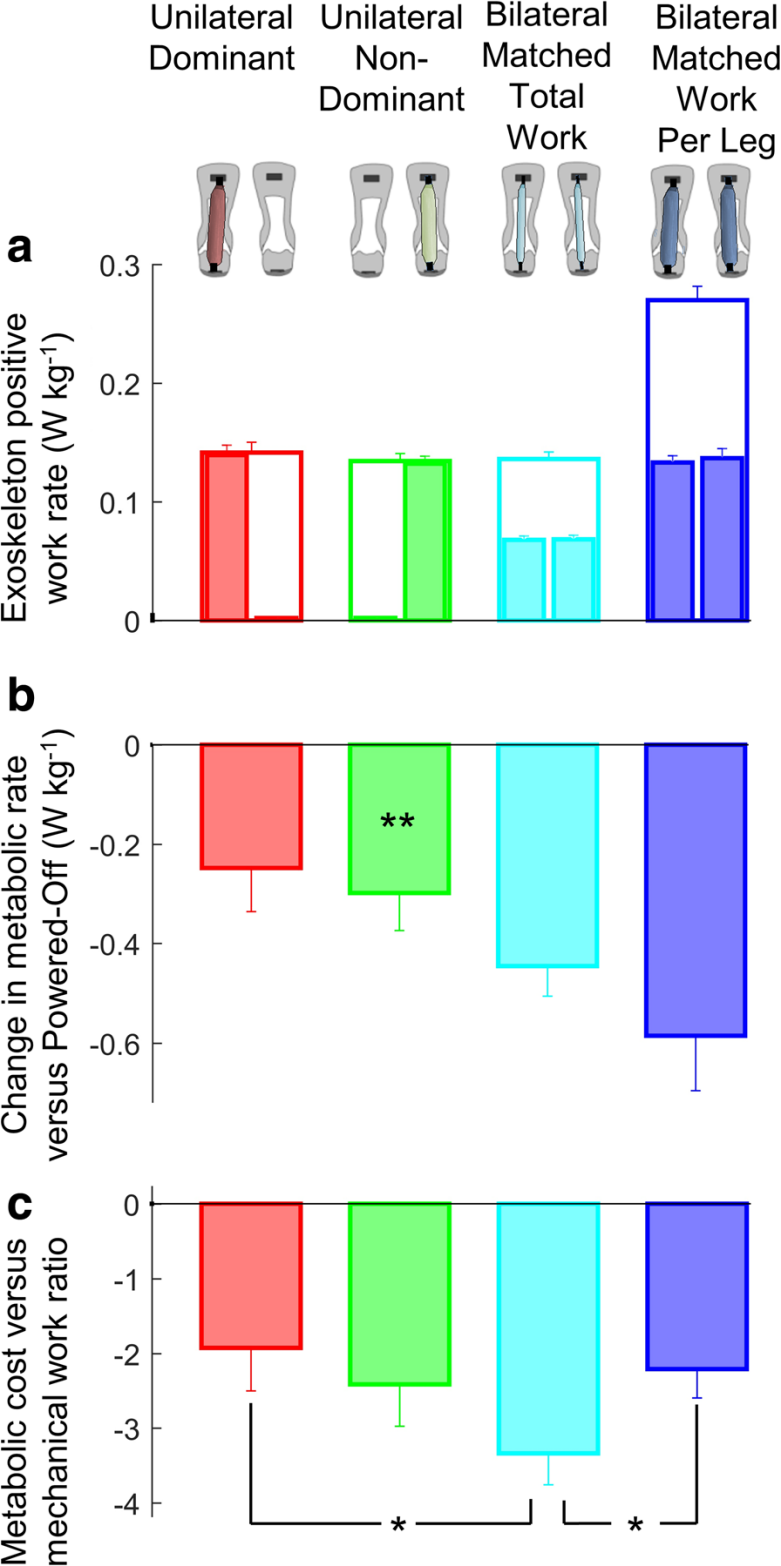
Exoskeleton and human energetics. **a** Exoskeleton positive work rate. Narrow filled bar plots represent the work rate per leg from the leg that is aligned on top of the figure. Empty wide bar plots represent the bilateral sum. **b** Change in metabolic rate versus the *Powered-Off* condition. **c** Metabolic cost versus mechanical work ratio. Colors represent the *Unilateral*, *Bilateral* and *Powered-Off* conditions indicated by figures on top of the chart columns. Error bars represent the standard error. Asterisks on top of the bar plots in panel b represent significant differences of *Unilateral* conditions versus the *Powered-Off* condition. Brackets indicate significant differences between powered conditions. Only the pairwise comparisons that are relevant for research questions 1 to 4 listed in the methods are analyzed (e.g., we did not compare the *Bilateral* conditions versus the *Powered-Off* condition since that was not relevant for the scope of this study). * is *p* ≤ 0.05, ** is *p* ≤ 0.01

Participants walked on a treadmill at 1.25 ms^− 1^ under different conditions. To isolate the effects of assistance asymmetry, we tested four assistance conditions with and without asymmetric assistance in which either the amount of assistance per leg was matched or the bilateral sum of the assistance was matched. In two *Unilateral* conditions, the participants wore both exoskeletons but only one leg was assisted. In one of these, the dominant leg was assisted, while in the other, the non-dominant leg was assisted; these conditions were called the *Unilateral Dominant* and *Unilateral Non-Dominant* conditions, respectively. Leg dominance was determined by asking for the preferred take-off leg for jumping. In the *Bilateral Matched Total Work* condition, half of the rate of work from the *Unilateral* conditions was applied to each leg such that the bilateral sum was equal to that of the *Unilateral* conditions. In the *Bilateral Matched Work Per Leg* condition, the same rate of work per leg as in the assisted leg of the *Unilateral* condition was provided to both legs such that the bilateral sum was double of that of the *Unilateral* conditions (Fig. 2a). In the *Powered-Off* condition, participants walked with exoskeletons on both legs but with no assistance.

### Exoskeleton control

A control program (LabVIEW, National Instruments, Austin, TX, USA) permitted the specification of the timing and the rate of positive work. For real-time control, exoskeleton power was calculated by multiplying the exoskeleton moment by the angular velocity of the ankle. The exoskeleton moment and angular velocity of the ankle were determined based on the displacement sensors and load cells. The rate of positive work assistance was calculated by averaging the positive exoskeleton power over a moving window of 10 strides. A learning algorithm similar to that used by [24, 42, 43] adjusted the supplied air pressure such that the desired rate of positive work assistance was maintained. In the *Unilateral* conditions and the *Bilateral Matched Work Per Leg* condition, we set the controller to provide approximately 0.13 W kg^− 1^ per assisted leg. In the *Bilateral Matched Total Work* condition, we set the controller to provide approximately 0.13 W kg^− 1^ for the sum of both legs. These chosen assistance levels were below the level where we found that metabolic rate stops decreasing further with additional assistance in a study with the same device [24]. The control program also triggered the start and end of the actuation at desired percentages of the stride based on the previous stride. The pneumatic muscles were triggered to assist from 44 to 62%. In two previous studies with the same exoskeleton we used an end timing of 62-63% and determined that an onset timing of 42-43% is optimal in combination with different assistance magnitudes [24, 26], therefore the used settings were close to the optimal actuation pattern for the current device.

### Protocol

Before the data collection the participants walked for about 5 min in each exoskeleton condition. The order of the conditions was randomized and the entire habituation lasted a total duration of 20 min based on an earlier study with the same exoskeleton that indicates that participants need on average about 20 min to adapt to exoskeleton walking [44]. During the actual experiment, participants walked for 4 min in each condition alternating with 2 min of rest. In a supplementary analysis in a study with the same exoskeleton we found that participants adapt quickly enough to changes in conditions such that metabolic rate reaches steady state after about 2 min [24]. The *Powered-Off* condition was conducted twice, and we calculated the average to improve our estimate of the metabolic baseline. The conditions were randomized. Due to hardware malfunctions and scheduling constraints, we did not complete 4 out of the 66 trials and treated these as missing values.

### Data collection

We measured breath-by-breath O_2_ consumption and CO_2_ production via indirect calorimetry (COSMED, K4b2, Rome, Italy). We estimated the metabolic rate of the last 2 min of each condition based on the protocol described in [45]. We calculated reductions in the metabolic rate by subtracting the metabolic rate from *Powered-Off*. We recorded kinematics at a rate of 200 frames per s for 10 s per condition with motion capture (Pro Reflex, Qualisys AB, Gothenburg, Sweden). Marker positions and pneumatic muscle force data were filtered with a Butterworth low-pass filter with a 12 Hz cut-off. We calculated sagittal joint angles using Visual3D (C-Motion, Germantown MD, USA). We also calculated exoskeleton moment by multiplying exoskeleton force from the load cell by the moment arm calculated with motion capture from markers on the pneumatic muscle attachments and the ankle joint. Next, we calculated exoskeleton power by multiplying the ankle angular velocity by the exoskeleton moment, and we calculated the rate of positive work assistance by integrating the positive power over time and dividing by the stride cycle duration. We estimated the center-of-mass position and velocity based on the pelvis markers [46]. We used a kinematic gait event detection algorithm to determine the timing of heel strike and toe off [47]. We calculated step length based on the gait event detection and treadmill speed. All timeseries data were stride-normalized based on the detected heel strike timings.

### Statistics

We used paired t-tests for specific comparisons of interest regarding mechanical, energetic and kinematic parameters. We compared (1) (the assisted leg in) the *Unilateral* conditions versus (the same leg in) the *Bilateral Matched Work Per Leg* condition to evaluate the direct effects of unilateral assistance; (2) (the unassisted leg in) the *Unilateral* conditions versus (the same leg in) the *Powered-Off* condition to evaluate the indirect effects on the unassisted leg; (3) the total effect on both legs in the *Unilateral* conditions versus the *Bilateral Matched Total Work* condition to evaluate the difference between focusing assistance on one leg versus distributing assistance over both legs; and (4) the total effect of the *Bilateral Matched Total Work* condition versus the *Bilateral Matched Work Per Leg* condition to assess the effects of assistance magnitude versus assistance symmetry. To compare the effects of assistance work rate asymmetry, the assistance work rate per leg and the total assistance work rate for both legs on the metabolic rate, we used a mixed-model ANOVA. We reported all descriptive results as the mean ± standard error.

## Results

### Exoskeleton mechanics

**Fig. 3:**
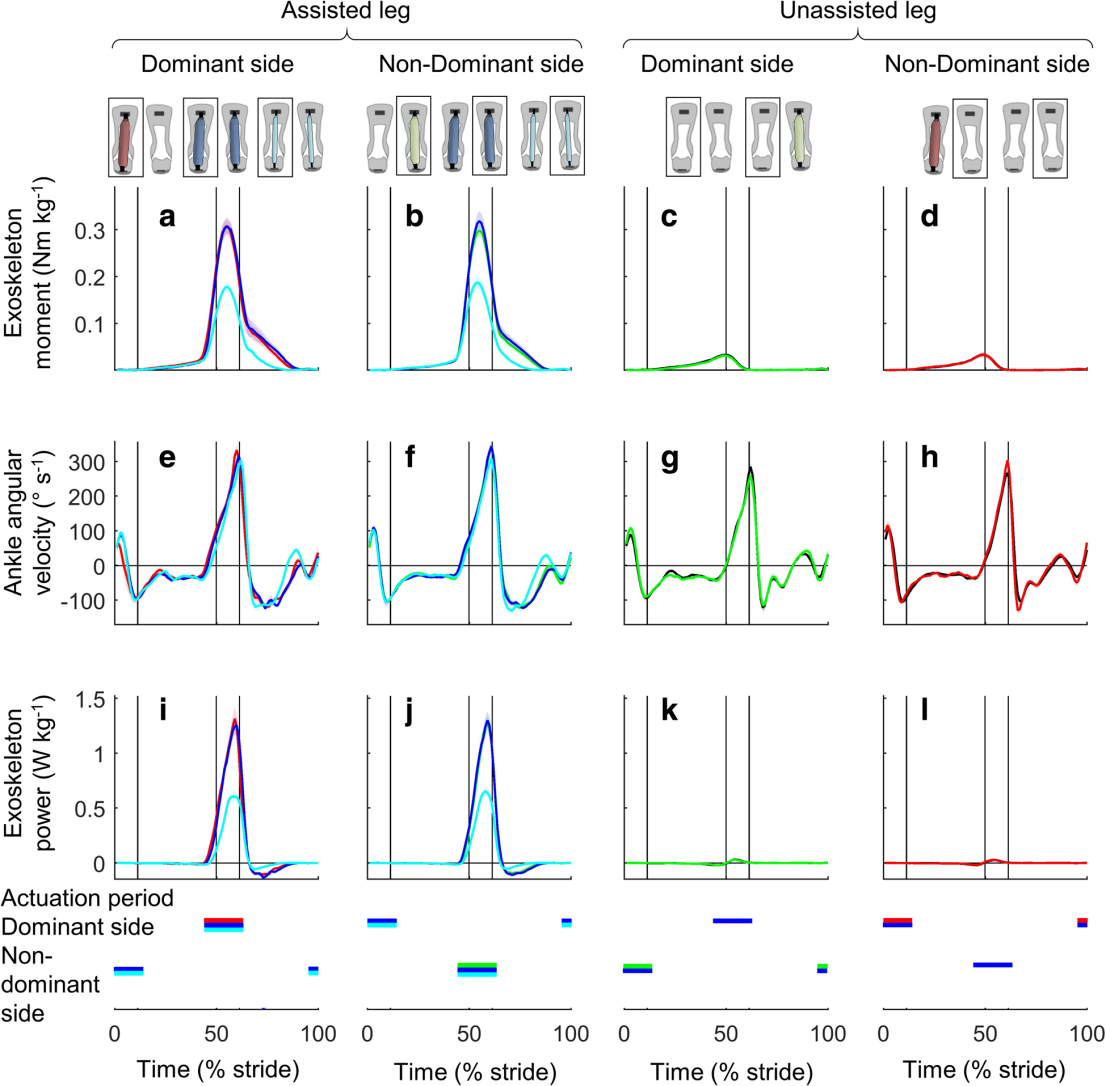
Actuation conditions. **a**-**d** Exoskeleton moment. **e**-**h** Ankle angular velocity. **i**-**l** Exoskeleton power. Left two columns show assisted leg. Right two columns show unassisted leg. Colored lines represent the *Unilateral*, *Bilateral* and *Powered-Off* conditions marked by rectangles in figures on top of the chart columns. Shaded area represents the standard error. Vertical lines show beginning and ending of single and double stance phases. Horizontal colored bars indicate the actuation period in conditions with corresponding colors

On average, in the *Unilateral* conditions, the exoskeleton provided 0.136 ± 0.008 W kg^− 1^ for the assisted leg. In the *Bilateral Matched Work Per Leg* condition, the exoskeletons provided 0.133 ± 0.006 W kg^− 1^ per leg (Figs. 2a and 3I-L). There was no significant difference in the exoskeleton work rate per assisted leg between the *Unilateral* and *Bilateral Matched Work Per Leg* conditions (*p*-value *Unilateral Dominant* versus *Bilateral Matched Work Per Leg* 0.403, *Unilateral Non-Dominant* versus *Bilateral Matched Work Per Leg* 0.276), indicating that we successfully matched the work rate per leg. In the *Bilateral Matched Total Work* condition, the exoskeletons provided 0.136 ± 0.007 W kg^− 1^ for the sum of both legs. There was no significant difference in the bilateral sum of the exoskeleton work rate for both legs between the *Unilateral* and *Bilateral Matched Total Work* conditions (*p*-value *Unilateral Dominant* versus *Bilateral Matched Total Work* 0.385, *Unilateral Non-Dominant* versus *Bilateral Matched Total Work* 0.682), indicating that we successfully matched the total work rate. The stride-average of exoskeleton moment for the assisted leg in the *Unilateral* conditions was 0.055 ± 0.004 Nm kg^− 1^ (Fig. 3a, b). The stride-average of exoskeleton moment in the *Bilateral Matched Work Per Leg* condition was 0.056 ± 0.004 Nm kg^− 1^. There was no significant difference in the stride-average of exoskeleton moment per assisted leg between the *Unilateral* and *Bilateral Matched Work Per Leg* conditions (*p*-value *Unilateral Dominant* versus *Bilateral Matched Work Per Leg* 0.106, *Unilateral Non-Dominant* versus *Bilateral Matched Work Per Leg* 0.125). The stride-average of exoskeleton moment in the *Bilateral Matched Total Work* condition was 0.032 ± 0.002 Nm kg^− 1^.

### Metabolic rate

Exoskeleton assistance reduced the metabolic rate in all conditions compared with that of the *Powered-Off* condition. Changes in metabolic rate compared with that of the *Powered-Off* condition were − 6.4 ± 3.3% for *Unilateral Dominant*, − 7.9 ± 2.2% for *Unilateral Non-Dominant*, − 11.3 ± 1.5% for *Bilateral Matched Total Work*, and − 14.9 ± 2.5% for *Bilateral Matched Work Per Leg* (Fig. 2b). The *Unilateral Non-Dominant* condition significantly reduced metabolic rate versus *Powered-Off* (*p*-value 0.003). The *Unilateral Dominant* condition trended towards reducing metabolic cost but this was not significant (*p*-value 0.066). We found the highest reduction in the *Bilateral Matched Work Per Leg* condition. The highest ratio of metabolic rate reduction versus positive work rate assistance was found in the *Bilateral Matched Total Work* condition (− 3.340 ± 0.416 W per W, Fig. 2c). This ratio was higher than that of the *Unilateral Dominant* condition (− 1.927 ± 0.722, *p*-value 0.048) and that of the *Bilateral Matched Work Per Leg* condition (*p*-value 0.020). Using a mixed-model ANOVA, we found that the change in metabolic rate is related to the work rate per assisted leg and the work rate difference between both legs according to the following formula:

$$ {\displaystyle \begin{array}{l} Change\ in\ metabolic\ rate\ \left(W\ {kg}^{\hbox{-} 1}\right)=\\ {}\hbox{-} 4.26\cdotp Work\ rate\  per\  assisted\  leg\ \left(W\ {kg}^{\hbox{-} 1}\right)+ 2.75\cdotp Absolute\ work\ rate\ difference\ \left(W\ {kg}^{\hbox{-} 1}\right)\end{array}} $$

(*p*-value Work rate per assisted leg is 3·10^-^^7^, *p*-value work rate difference is 3·10^− 4^, Additional file 1).

### Spatiotemporal results

**Fig. 4:**
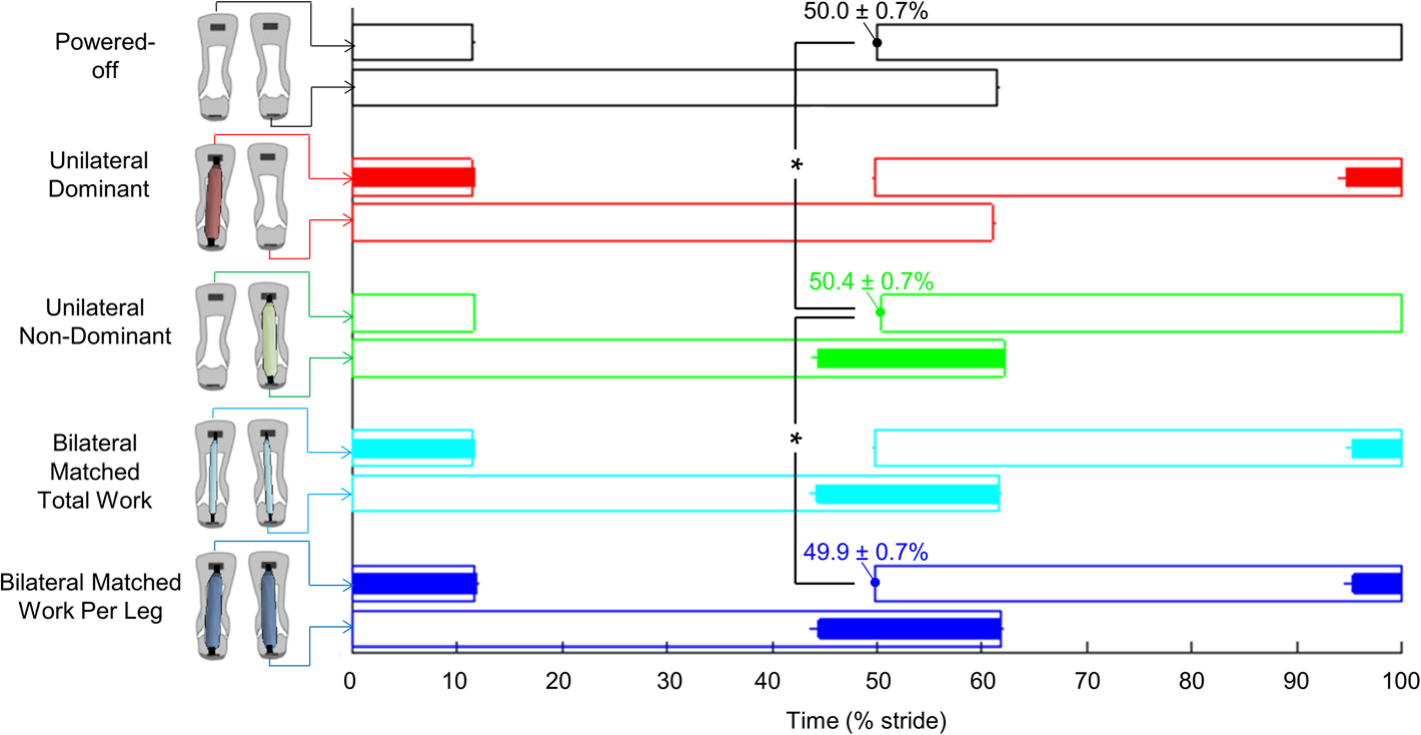
Spatiotemporal results. Horizontal axis indicates right-side stride cycle percentage. Empty bars represent the stance phase duration. Filled bars represent the assistance phase. Numbers indicate timing of opposite leg heel contact of conditions that show significant differences. Colors represent the *Unilateral*, *Bilateral* and *Powered-Off* conditions, and each horizontal line corresponds to the leg that is shown in the figures on the left. Error bars represent the standard error. Brackets represent significant differences between conditions. Only the pairwise comparisons that are relevant for the research questions listed in the methods are analyzed. * is *p* ≤ 0.05

In the *Unilateral Non-Dominant* condition, the heel contact of the unassisted leg occurred 0.5 ± 0.2% later than in the *Powered-Off* condition (*p*-value 0.013) and 0.6 ± 0.2% later than in the *Bilateral Matched Work Per Leg* condition (*p*-value 0.014, Fig. 4). On average, in the *Unilateral Dominant* condition, the heel contact of the unassisted leg also occurred 0.4 ± 0.3% later than in the *Powered-Off* condition, but this difference was not significant (*p*-value 0.250). No significant differences were found in the step frequency or duty factor among the *Unilateral*, *Bilateral* and *Powered-Off* conditions.

### Joint kinematics

**Fig. 5:**
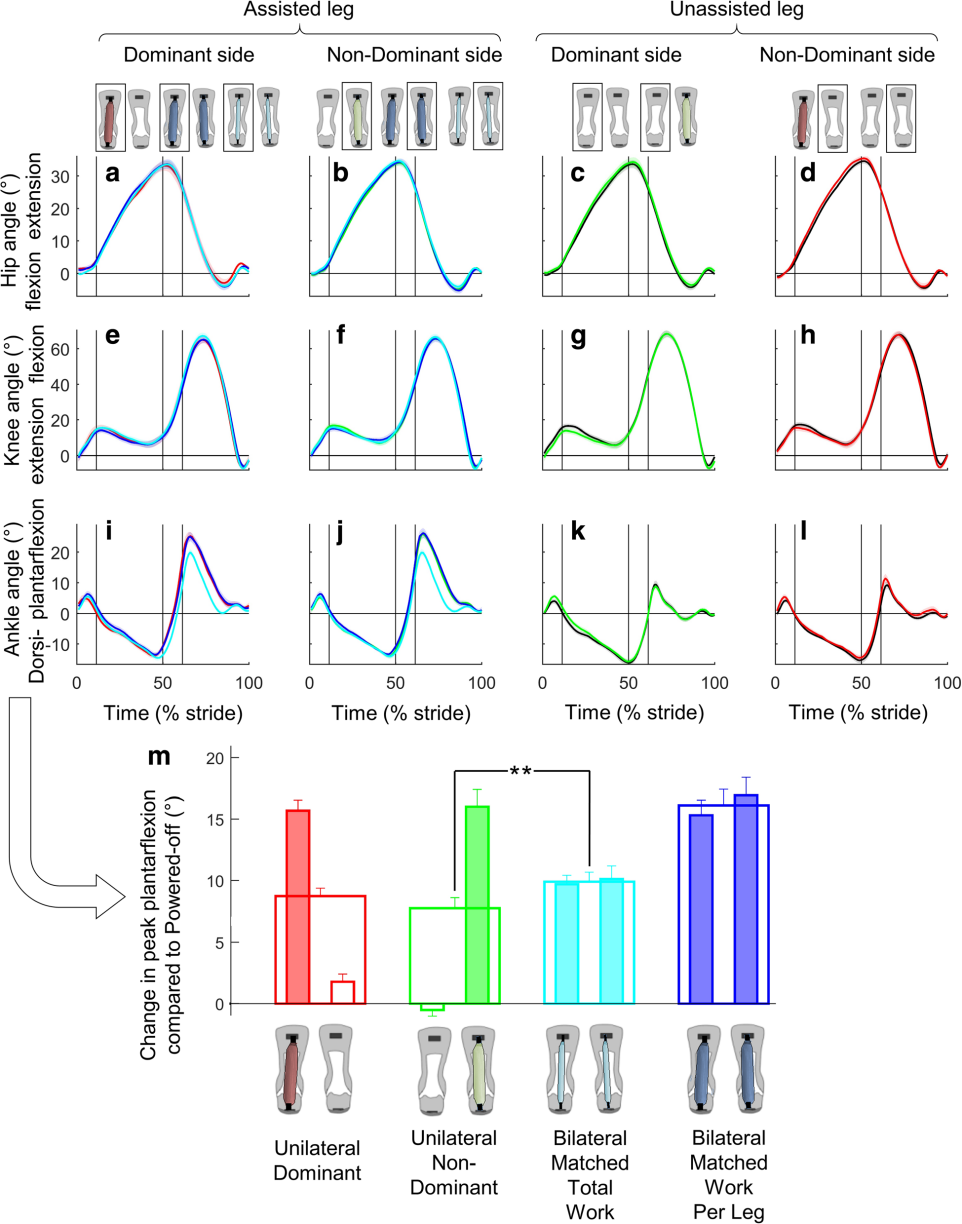
Joint kinematics. **a**-**d** Hip. **e**-**h** Knee. **i**-**l** Ankle. Left two columns show assisted leg. Right two columns show unassisted leg. Colored lines are from the *Unilateral*, *Bilateral* and *Powered-Off* conditions marked by rectangles in figures on top of the chart columns. Shaded area represents the standard error. Vertical lines show beginning and ending of single and double stance phases. **m** Change in peak plantarflexion compared with *Powered-Off*. Narrow filled bar plots represent peak plantarflexion per leg from the leg that is aligned in the figure below. Empty wide bar plots represent the bilateral mean. Error bars represent the standard error. Brackets represent significant differences between powered conditions. Only the pairwise comparisons that are relevant for the research questions listed in the methods are analyzed. ** is *p* ≤ 0.01

There were no significant differences in peak plantarflexion between the individual legs when comparing the same leg between the *Unilateral* and *Bilateral Matched Work Per Leg* conditions. However, the change in peak plantarflexion in the *Bilateral Matched Total Work* condition trended to be larger than the average of the assisted and unassisted legs in the *Unilateral* conditions (*p*-value *Unilateral Non-Dominant* versus *Bilateral Matched Total Work* 10^− 4^, *Unilateral Dominant* versus *Bilateral Matched Total Work* 0.063^− 4^, Fig. 5m). There were no other relevant significant differences in the evaluated peak joint angles of the ankle, knee or hip among the *Unilateral*, *Bilateral* and *Powered-Off* conditions (Fig. 5a-l).

### Center-of-mass kinematics

**Fig. 6:**
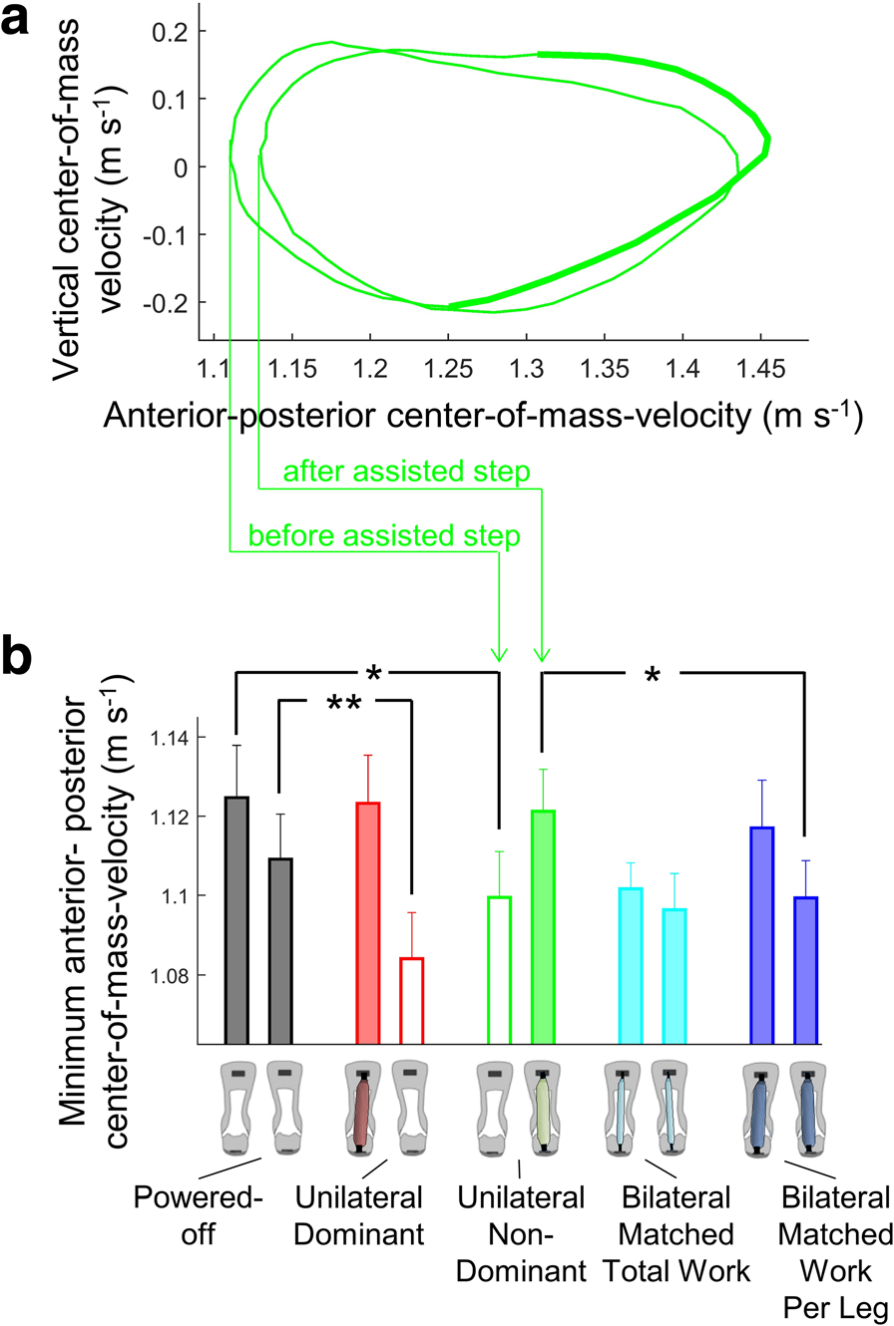
Center-of-mass kinematics. **a** Vertical center-of-mass velocity plotted versus horizontal center-of-mass velocity from the *Unilateral Non-Dominant* condition. Wider part of the line represents the assisted portion of the center-of-mass trajectory. **b** Minimum anterior-posterior center-of-mass velocity. Narrow empty bar plots represent the minimum velocity during the unassisted leg stance. Narrow filled bar plots represent the minimum velocity during the assisted leg stance. Empty wide bar plots represent the bilateral mean. Error bars represent the standard error. Brackets represent significant differences between powered conditions. Only the pairwise comparisons that are relevant for the research questions listed in the methods are analyzed. * is *p* ≤ 0.05, ** is *p* ≤ 0.01

The minimum horizontal center-of-mass velocity before the heel strike of the assisted leg was lower in the *Unilateral* conditions than in the *Powered-Off* condition (*p*-value *Unilateral Dominant* versus *Powered-Off* 0.002, *Unilateral Non-Dominant* versus *Powered-Off* 0.022, Fig. 6). In the *Unilateral Non-Dominant* condition, the minimum horizontal velocity after the heel strike of the assisted leg was also lower than that in the *Bilateral Matched Work Per Leg* condition (*p*-value 0.017).

## Discussion

The aim of our study was to compare the effects of unilateral and bilateral exoskeleton assistance in a within-subject design. On average the *Unilateral* conditions reduced the metabolic rate by 7% compared with the *Powered-Off* condition (Fig. 2). As expected, the highest absolute reduction was found in the bilateral condition with the highest assistance (*Bilateral Matched Work Per Leg*). However, we found the highest ratio of metabolic cost reduction versus positive work in the bilateral condition with low assistance per leg (*Bilateral Matched Total Work*), which indicates that a given amount of exoskeleton assistance is the most efficiently used when it is evenly distributed over both legs.

Based on the literature, it was uncertain whether unilateral assistance would reduce the metabolic rate. The trends from simulations [4], measurements in populations with push-off asymmetry [48, 49] and experiments that evoke gait asymmetry in healthy participants [11, 41] indicate that push-off asymmetry can lead to energy losses and increased metabolic rates. An exoskeleton study with unilateral plantarflexion assistance had conditions that reduced the metabolic rate but also conditions that increased the metabolic rate by up to 22% compared with the *Powered-Off* condition [50]. In our experiment, the *Unilateral* conditions reduced the metabolic rate with a ratio of 2.2 W per W positive work rate. This ratio is close to the efficiency ratio of 2.5 found in the other unilateral exoskeleton study [50] and falls within the reported range for bilateral exoskeletons (1.6 [51] to 4.7 [26]).

A possible explanation for why the *Unilateral* conditions did not increase the metabolic rate compared with the *Powered-Off* condition could be that they caused only slight gait asymmetry (Additional file 2). The *Unilateral* conditions did not cause significant asymmetry in step frequency (Fig. 4), which could otherwise have led to an increased metabolic rate [41]. We only observed effects of assistance asymmetry in joint kinematics at the ankle (Fig. 5). Similarly, Wutzke et al. [11] found no significant kinematic asymmetry in any joint except the ankle during walking with unilateral ankle restriction. It seems that healthy participants can retain normal kinematics despite strong perturbations. We also did not find indications of increased spatiotemporal variability or increased imbalance in the *Unilateral* conditions (Additional file 3). This echoes findings from split-belt walking studies showing that healthy participants can walk comfortably even with large differences in belt speed [52, 53].

We observed asymmetry in the timing of opposite heel contact, but this asymmetry was less than 1%. The slower forward center-of-mass velocity during the assisted leg stance and faster velocity during the unassisted leg stance (Fig. 6) correspond to findings from simulations and experiments performed by Adamczyk and Kuo [4] with unilateral amputees. It is possible that this adaptation allows participants to save energy by allowing the exoskeleton to accelerate the center off mass during the assisted step and by saving effort during the unassisted step. A follow-up analysis of joint kinetics and EMG data would allow to interpret where the metabolic savings could come from and more specifically if some of the metabolic savings come from the unassisted leg in addition to the assisted leg.

We found the highest metabolic rate versus mechanical work ratio in the *Bilateral Matched Total Work* condition. The metabolic cost reduction resulting from assisting both legs with low assistance was higher than the average metabolic cost reduction resulting from the *Unilateral* conditions. We found a similar “bilateral surplus” effect (i.e., the opposite of “bilateral deficit” as in [54]) in peak plantarflexion. More specifically, we found that the increase in peak plantarflexion in the *Bilateral Matched Total Work* condition was higher than the mean of the assisted and unassisted leg in the *Unilateral* conditions.

The question remains why the assistance was the most efficient in the *Bilateral Matched Total Work* condition. Is it because of the low assistance asymmetry, or is it because of the assistance work rate per leg or the total assistance work rate for both legs? Using a mixed-model ANOVA with stepwise elimination, we found that the assistance work rate per assisted leg and the assistance work rate difference between both legs are more determining than the total assistance work for both legs (Additional file 1). This information could be used to bridge the gap between studies with unilateral and bilateral exoskeletons. For example, Jackson and Collins [50] found metabolic rate reductions of up to 0.16 W kg^− 1^, or 5%, compared with the *Powered-Off* condition with a unilateral exoskeleton that provided a rate of 0.20 W kg^− 1^ positive work. This value is similar to our reduction of 7% found for the *Unilateral* conditions. Based on the assistance work asymmetry coefficient from the mixed-model ANOVA, we can estimate that the optimal condition in the study by Jackson and Collins could have resulted in a reduction of 0.86 W kg^− 1^, or 28%, if they provided an additional assistance of 0.20 W kg^− 1^ to the other leg. This estimation is slightly higher than the best results recently obtained from bilateral exoskeletons (− 21% in [24], − 23% in [28]); however, this estimation assumes perfect assistance symmetry, which is difficult to achieve. Furthermore, this estimation does not account for potential interaction effects with timing and magnitude parameters of the actuation pattern and potential different effects depending on exoskeleton designs or control methods that are used. Therefore, we believe that the coefficient for the effect of assistance asymmetry that we found can only be used for rough estimates when applying it on other datasets than the dataset of the present study. The assistance asymmetry coefficient could also be used to estimate the losses in metabolic cost reduction due to assistance asymmetry in studies with bilateral exoskeletons.

A limitation of the interpretation of the coefficients for the effects of assistance work rate per leg and assistance work rate asymmetry that we found is that these coefficients were obtained from a dataset with only three work rate levels (*Powered-Off*, 0.068 and 0.135 W kg^− 1^) and only two assistance work rate asymmetry levels (no asymmetry and 100% asymmetry). It is likely that the effect of assistance asymmetry is smaller around low asymmetry levels. Another limitation is that the timing that was used was optimized for bilateral assistance. It could be that the smaller metabolic cost benefits from the *Unilateral* conditions are due to sub-optimal timing. To facilitate switching between conditions and to isolate the effect of assistance asymmetry from exoskeleton weight, we let the participants wear exoskeletons on both legs in the *Unilateral* conditions. It is uncertain if the effect of assistance asymmetry would have been smaller or larger if participants had worn only one exoskeleton in the *Unilateral* conditions. We did not calculate if any of the exoskeleton conditions had a net metabolic cost benefit compared to walking without exoskeleton. In previous studies we found that wearing the same type of bilateral exoskeleton while powered-off caused a metabolic penalty of 11% [24, 26]. Therefore, we can estimate that unilateral assistance while wearing both exoskeletons would increase metabolic cost by 4% compared to not wearing the exoskeleton and unilateral assistance while wearing only one exoskeleton would reduce metabolic cost by about 1.5% (assuming that the metabolic cost penalty for wearing a unilateral exoskeleton powered-off is half of the metabolic cost penalty for wearing a bilateral exoskeleton powered-off).

As expected, our participants had a symmetric walking pattern in the *Powered-off* condition. Therefore, it is logical that we found a positive coefficient for the effect of assistance work rate asymmetry, indicating that increasing the amount of assistance asymmetry is detrimental in healthy participants. Based on our data we do not know what would happen in patients who start off with an initial asymmetry such as hemiparetic stroke patients [5, 7–9]. Studies with a unilateral exoskeleton [21] and unilateral exosuits [20] indicate that unilateral assistance to the impaired leg can reduce the metabolic cost in stroke patients. However, we do not know exactly what level of assistance asymmetry is optimal. Awad et al. [20], found a correlation between improvement in propulsion symmetry and reduction in metabolic cost from an exosuit which suggests that tuning the assistance to reduce gait asymmetry as much as possible could be optimal. A follow-up analysis from the same group showed that reduction in metabolic cost was mostly correlated to center-of-mass power from the non-paretic limb [55]. Maybe, assisting stroke patients at the non-paretic side in addition to the paretic side could result in even greater reductions in metabolic cost? Similar protocols as our present study that compare different types of unilateral and bilateral assistance in patient populations with asymmetric gait could answer such questions.

## Conclusion

We found that *Unilateral* exoskeleton assistance can reduce the metabolic rate by 7 ± 3% compared with wearing a *Powered-Off* exoskeleton. Similar to results from walking with a unilateral prosthesis, we found slower forward center-of-mass velocity during the assisted leg stance and faster velocity during the unassisted leg stance, which is possibly an adaptation to reduce effort. We found the highest ratio of metabolic cost reduction versus positive work with *Bilateral Matched Total Work.* The results indicate that assistance work rate symmetry and the assistance work rate per leg are more important for metabolic cost reduction than the bilateral work rate sum. These results can help explain differences between studies with unilateral and bilateral exoskeletons and inform how unilateral assistance can potentially be used for practical applications, such as reducing gait asymmetry.

## Additional files

Additional file 1: Analysis of effect of total work, work per assisted leg and work asymmetry on change in metabolic rate. (PDF 43 kb)

Additional file 2: **Movie 1.** *Unilateral* condition. Participant walking on treadmill with assistance to the right leg and no assistance to the left leg. It can be qualitatively observed that there is little asymmetry in the gait pattern except in the ankle as shown quantitively in Figs. 4 and 5. (MP4 1264 kb)

Additional file 3: Spatiotemporal stability and balance results. A) Step width was calculated based on the average distance in the medio-lateral direction between the markers on the left and right foot during consecutive stance phases. B) Step width variability was calculated by taking the standard deviation of step width across different strides. C) Step length variability was calculated by taking the standard deviation of step time multiplied by treadmill speed across different strides. Bars represent population averages. Error bars represent the standard error. Colors represent the Unilateral, Bilateral and Powered-Off conditions shown in the figures on the bottom. Brackets represent significant differences between conditions. Only the pairwise comparisons that are relevant for the research questions listed in the methods are analyzed. ** is *p* ≤ 0.01. (TIF 2283 kb)
