# Supplementary material for: Exoskeleton assistance symmetry matters: unilateral assistance reduces metabolic cost, but relatively less than bilateral assistance
Source: J Neuroeng Rehabil. 2018 Aug 9;15:74. doi: 10.1186/s12984-018-0381-z (PMC6085709; doi:10.1186/s12984-018-0381-z)
Supplement: Supplementary file 1 — Analysis of effect of total work, work per assisted leg and work asymmetry on change in metabolic rate. (PDF 43 kb) [file 12984_2018_381_MOESM1_ESM.pdf]

Analysis of effect of total work, work per assisted leg and work asymmetry on change in metabolic rate

Statistical analysis: Mixed-model ANOVA

Fixed effects:

- Total work rate from both legs.
- Work rate per assisted leg: For the *Bilateral* and *Powered-Off* conditions, this value was entered as the average work from both legs. For the *Unilateral* conditions, this value was entered as the average from both legs. We had to use a single term for both legs because if we entered the work for the left and right legs, then intercorrelation problems would occur with the total work rate term.
- Absolute work rate difference: The absolute value of the difference in work rate between both legs. In the *Unilateral* conditions, this value is the difference in the work rate between the assisted and unassisted legs. In the *Bilateral* and *Powered-Off* conditions, this value was any small difference that happened to occur due to imperfections in the control and hardware performance. We could have chosen another asymmetry parameter (e.g., asymmetry index). The reason we selected the absolute work rate difference between legs is because this has the same unit as the other two fixed-effect parameters (i.e., W kg^{-1}) such that the coefficients can be compared in a meaningful way.

Random effect: Participants.

Outcome: Change in metabolic rate versus *Powered-Off.*

Stepwise elimination method: Backward elimination.

Criterion: Least significant term is removed on every iteration.

First iteration result: (Symbols indicate p-values: ** is p ≤ 0.01, * is p ≤ 0.05, ^{ns} is p > 0.05.)

Change in metabolic rate (W kg^{-1}) =

- 6.22·Work rate per assisted leg (W kg^{-1}) ^{ns} + 3.73·Absolute work rate difference (W kg^{-1}) ^{ns}

+ 0.99·Total work rate from both legs (W kg^{-1}) ^{ns} - 0.06 ^{ns}

Adjusted $R^2 = 0.48$

Final iteration result:

Change in metabolic rate (W kg^{-1}) =

*-4.26·Work rate per assisted leg (W kg^{-1}) ** + 2.75·Absolute work rate difference (W kg^{-1}) ***

Adjusted $R^2 = 0.49$

Interpretation:

The parameter for the total work rate was eliminated by the stepwise elimination, while the work rate per assisted leg and the work rate difference significantly contributed to the estimated change in metabolic rate. This indicates that in this dataset, the way in which the total work is distributed over both legs is more important for explaining changes in metabolic rate than the bilateral sum of the work assistance for both legs. In addition, it makes sense that the constant was eliminated because one would expect the fit to pass through zero. The resulting coefficients seem logically meaningful: The coefficient for the absolute work rate difference is positive, which makes sense because we assume that asymmetry is detrimental for reducing the metabolic rate. In the case of symmetrical bilateral assistance (i.e., the work rate difference term is zero), a coefficient for the work rate per assisted leg of -4.26 would result in a ratio of metabolic rate versus bilateral mechanical work rate of -2.13 W per W, which falls within the range of previously published coefficients (from -1.6 [51] to -4.7 [26]). The fact that the absolute value of the coefficient for the work rate per assisted leg is larger than the coefficient for work rate asymmetry means that even with 100% asymmetry (i.e., all assistance delivered to one leg and no assistance on the other leg), there is still a metabolic reduction.
