# Supplementary figures and images for: Exoskeleton assistance symmetry matters: unilateral assistance reduces metabolic cost, but relatively less than bilateral assistance
Source: J Neuroeng Rehabil. 2018 Aug 9;15:74. doi: 10.1186/s12984-018-0381-z (PMC6085709; doi:10.1186/s12984-018-0381-z)

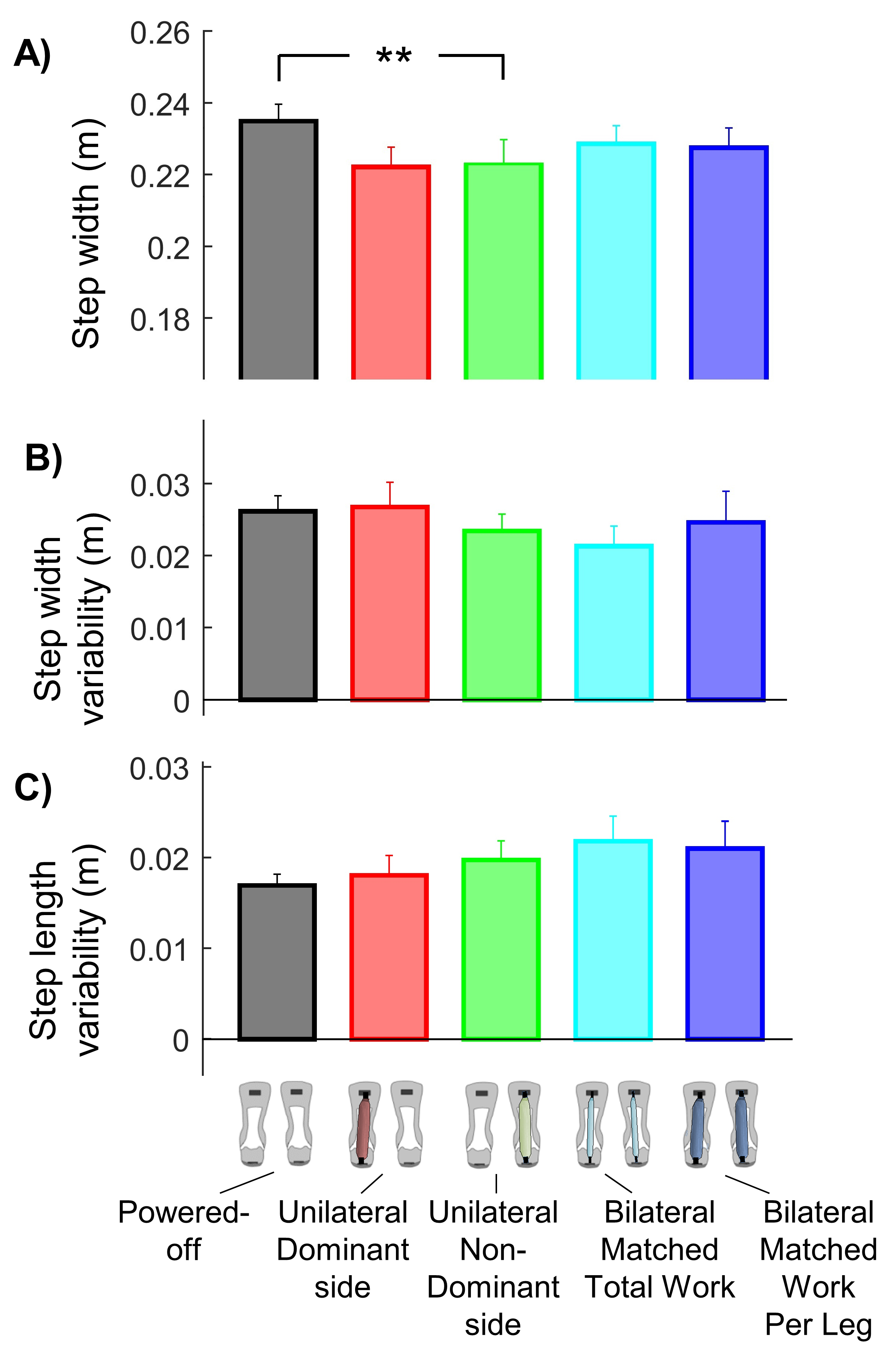

Supplement: Supplementary file 3 — Spatiotemporal stability and balance results. A) Step width was calculated based on the average distance in the medio-lateral direction between the markers on the left and right foot during consecutive stance phases. B) Step width variability was calculated by taking the standard deviation of step width across different strides. C) Step length variability was calculated by taking the standard deviation of step time multiplied by treadmill speed across different strides. Bars represent population averages. Error bars represent the standard error. Colors represent the Unilateral, Bilateral and Powered-Off conditions shown in the figures on the bottom. Brackets represent significant differences between conditions. Only the pairwise comparisons that are relevant for the research questions listed in the methods are analyzed. ** is p ≤ 0.01. (TIF 2283 kb) [file 12984_2018_381_MOESM3_ESM.tif]
